# LBW and SGA Impact Longitudinal Growth and Nutritional Status of Filipino Infants

**DOI:** 10.1371/journal.pone.0159461

**Published:** 2016-07-21

**Authors:** Rachel A. Blake, Sangshin Park, Palmera Baltazar, Edna B. Ayaso, Donna Bella S. Monterde, Luz P. Acosta, Remigio M. Olveda, Veronica Tallo, Jennifer F. Friedman

**Affiliations:** 1 The Warren Alpert Medical School of Brown University, Providence, RI, United States of America; 2 Center for International Health Research, Rhode Island Hospital, The Warren Alpert Medical School of Brown University, Providence, RI, United States of America; 3 Research Institute for Tropical Medicine, Manila, Philippines; 4 Remedios Trinidad Romualdez Hospital, Tacloban City, Leyte, The Philippines; Telethon Institute for Child Health Research, AUSTRALIA

## Abstract

We performed this study to longitudinally compare rates of stunting, wasting and underweight among low birthweight (LBW), non-LBW, and/or small-for-gestational age (SGA) and non-SGA infants in Leyte, The Philippines and factors that predicted catch up. Birthweights of 357 infants born in Leyte, The Philippines were obtained within 48 hours of delivery and infants were evaluated at one, six and 12 months. Newborns were classified as LBW, SGA, or both. We derived length-for-age, weight-for-length and weight-for-age Z-scores using WHOAnthro. Generalized estimating equations models were used to compare the differences in prevalence and mean Z-scores for these growth and nutritional outcomes, with separate models made with LBW and SGA as distinct primary predictors. We compared the longitudinal risk of stunting, wasting and underweight during infancy among LBW versus non-LBW and SGA versus non-SGA infants, while also evaluating key potential confounding, explanatory and modifying covariates. Overall, 9.0% of infants were born prematurely, 14.0% of infants were LBW and 22.9% were SGA. LBW infants had significantly increased odds of stunting, wasting and underweight persisting to 12 months of age, and SGA infants had significantly increased odds of stunting and underweight. LBW and SGA infants had higher rates of weight-for-length gain in the first month of life. Maternal educational attainment and exclusive breastfeeding decreased the risk of stunting and undernutrition. In this setting, LBW and SGA infants have higher rates of growth stunting and undernutrition during the first year of life and do not exhibit catch-up growth by 12 months of age.

**Clinical Trial Registration:**

NCT00486863

## Introduction

In low resource settings, undernutrition during infancy is thought to greatly increase the risk of infant and early childhood mortality [1]. Poor growth during infancy has been shown to result in increased risk of short stature among adults, which is associated with decreased work productivity and higher rates of adverse birth outcomes for women of reproductive age [2].

Studies have demonstrated that low birthweight (LBW) and small-for-gestational age (SGA) are risk factors for both linear growth stunting and undernutrition among young children [3]. Specifically, prospective studies conducted in Cebu, The Philippines demonstrated that LBW infants are at higher risk of stunting for the first two years of life than normal birthweight infants, with the greatest effect during the first year of life [3]. LBW has additionally been associated with other adverse health outcomes during infancy and adulthood in low, middle and high-income nations [4–8].

The prevalence of LBW deliveries ranges from 12–25% in low-income nations compared to 7% in higher-income nations. In affluent populations, most LBW infants are born premature, while in low-income countries the majority are full-term infants who have experienced growth restriction *in utero*, often culminating in an SGA newborn [6, 9, 10]. Thus, understanding the post-natal growth consequences of SGA and factors modifying this relationship is of great importance in the low and middle income country (LMIC) context.

A significant challenge to our understanding of the influence of SGA on post-natal morbidity and growth trajectories is that these newborns represent a heterogeneous group. Specifically, most studies define SGA as a birthweight that is less than the 10^th^ percentile of a healthy reference curve for the newborn’s sex and gestational age (GA). SGA may occur, however, due to the pathologic process of intrauterine growth restriction (IUGR), whereby a fetus does not reach its *in utero* growth potential, or as a result of normal variability whereby a fetus achieves its *in utero* growth potential, which is constitutionally small. The availability of the International Fetal and Newborn Growth Consortium for the 21^st^ Century standard (INTERGROWTH-21^th^) to determine SGA, rather than reliance on United States derived reference curves, has been demonstrated to decrease the percentage of newborns categorized as SGA [11, 12]. This approach may better capture SGA that is due to a pathologic process in the LMIC setting.

The primary objectives of this study were to compare rates of stunting, wasting and underweight at one, six and 12-months-old among LBW, non-LBW, SGA and non-SGA infants born in Leyte, The Philippines. Additional objectives included i) assessment of differences in growth velocity across birthweight status groups during specific age “windows,” ii) determining the timing of growth and nutritional catch-up where this occurred, and iii) elucidating other risk factors (mode of feeding, maternal nutritional status and educational attainment) for growth faltering and undernutrition during infancy. Quantification of these relationships may further emphasize the need for prenatal interventions to reduce the risk of LBW and SGA births, as well as post-natal interventions to optimize catch-up growth and nutrition in resource poor settings.

## Methods

### Study Population

**Fig 1:**
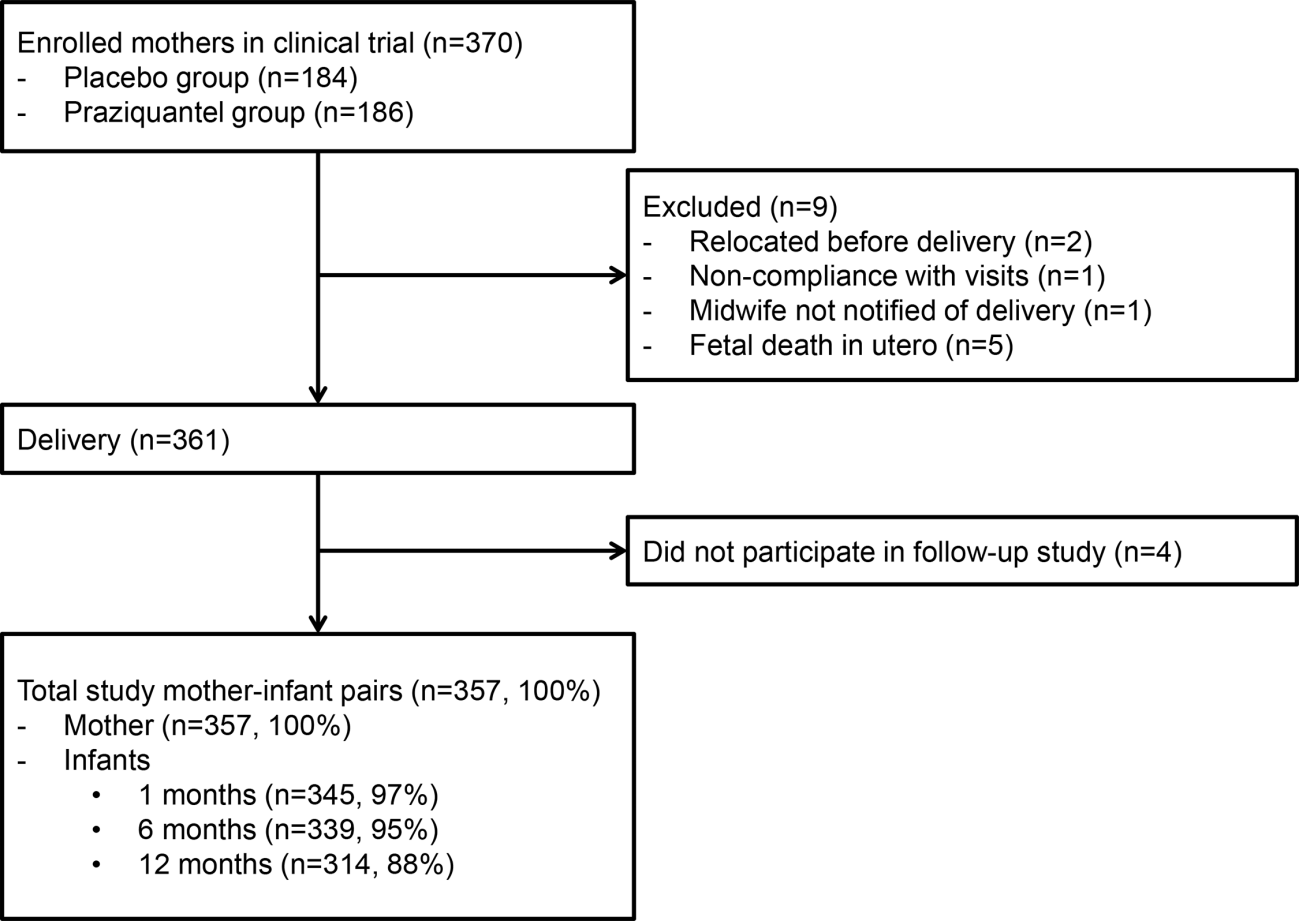
Flow of participants.

This study utilizes data collected as part of an NIH-funded double blind randomized controlled trial (RCT) of Praziquantel given at 12–16 weeks gestation (ClinicalTrials.gov, NCT00486863) and a Thrasher Fund supported study of the infants born to these women. The RCT enrolled 370 otherwise healthy pregnant women from rice farming villages in northeastern Leyte, The Philippines with singleton pregnancies who were infected with *Schistosomiasis japonicum* as described [13]. As part of screening procedures for enrollment, women underwent a transabdominal ultrasound to determine GA, viability of fetus and singleton pregnancy. Maternal anthropometric measures were also made in the first-trimester and used for statistical analyses. Women were deemed healthy and eligible to participate based on history, physical examination, and laboratory studies. Women were randomized 1:1 to placebo or Praziquantel. All women were provided with prenatal vitamins with iron at enrollment and reported compliance was 99.7%. Malaria is not endemic and the prevalence of HIV is <0.1% [14]. Of note, Praziquantel did not significantly impact birthweight or risk of LBW or SGA (manuscript in review), such that this was not included in these post-natal analyses. All newborn live births (n = 357) were eligible to participate (Fig 1). At the time of close out from the NIH trial, when the newborn was 28 days of age, mothers were asked to enroll in this separate follow-up study of their infants.

### Newborn Measures

As part of the study protocol, all mothers gave birth at a municipal health center or were referred to Remedios Trinidad Romualdez Hospital if indicated. Newborns were weighed within 48 hours of delivery on a Tanita model BD-585 portable scale (Arlington Heights, MD) accurate to 10g, and all birthweights with the exception of one were obtained within 24 hours. LBW was defined as weight <2.5 kg and prematurity as birth <37 weeks gestation. SGA was defined as birthweight <10^th^ percentile for GA using the INTERGROWTH-21^th^ [11]. Ultrasound derived GA was used to determine prematurity and SGA status.

### Infant Follow-Up

Infants were assessed at RTR hospital at one, six and 12-months-old. They were seen by the study pediatrician who conducted a history, physical examination and assessed length, weight and head circumference. Recumbent length was measured using a pediatric stadiometer (Ellards Instrumentation LTD, Monroe, WA) as per Gibson [15]. WHOAnthro was used to derive length-for-age (LAZ), weight-for-length (WLZ) and weight-for-age (WAZ) Z-scores at one, six and 12-months-old [15, 16]. Stunting, wasting and underweight were defined as LAZ, WLZ or WAZ <-2.0, respectively.

### Assessment of Potential Confounders and Modifying Covariates

Maternal educational status was defined as having attained a high school degree or greater level of education versus less than high school completion. At the one, six and 12-month follow-up visits, mothers were asked about infant feeding practices, which were categorized as exclusive breastfeeding, bottle feeding or mixed.

### Statistical Analyses

Group differences (LBW vs. non-LBW; SGA vs. non-SGA) were determined by using a Student’s *t* test or Wilcoxon rank-sum test for continuous variables and Fisher’s exact or Chi-square test for categorical variables. To assess the effects of LBW and SGA on LAZ, WLZ, and WAZ and length, weight-for-length, and weight gains, as well as the probability of stunting, wasting and underweight, we implemented generalized estimating equations (GEE) models with an exchangeable correlation structure and robust standard error estimation. This approach was used to compare differences in growth and nutritional parameters longitudinally during infancy, comparing infants born in distinct birthweight categories while adjusting for within-subject correlation. These models capture distinct outcomes (eg, stunting) at three timepoints for each infants and relate each of these outcomes to time varying predictors at the corresponding timepoint, as well as non-time varying covariates such as sex. Finally, we employed GEE models to assess the effects of LBW and SGA on absolute gains in length and weight as well as weight for weight-for-length in the first year of life.

GEE models were also used to identify other risk factors for adverse growth and nutritional outcomes as well as effect modifiers of the relationship between birthweight category and risk of stunting, wasting and underweight throughout infancy. Distinct models with LBW or SGA as the primary predictor were evaluated for each of the nutritional outcome measures, as we could not include the non-independent variables of SGA/LBW in the same models. LBW and SGA status (in distinct models) and potential confounders with *P* values <0.2 in the univariable models were considered for inclusion in multivariable models. Multivariable models for stunting, wasting and underweight were built by performing a backward elimination process until only significant variables remained.

All statistical analyses were performed using SAS 9.4 (SAS Institute Inc., Cary, NC). *P* values <0.05 were considered to be significant, except in univariable analyses.

### Ethical Considerations

Infants with an acute or chronic medical condition or malnutrition diagnosed during the newborn period or during the infant follow-up study were referred for care. The pregnancy trial and the infant follow-up studies were separately approved by both the Rhode Island Hospital Institutional Review Board in Providence, RI and the Ethics Review Board of the Research Institute of Tropical Medicine in Manila, The Philippines. All maternal participants of the study provided written informed consent approved by both review boards.

## Results

Of 370 pregnant women enrolled, there were five fetal deaths *in utero*. Birthweight was ascertained for 361 of 365 live births and 357 infants enrolled in the infant follow up study. Of these, 14.0% were LBW and 22.9% were SGA (Table 1). Approximately 80.0% of LBW infants were SGA and 48.8% of SGA infants were LBW. There were 32 infants (9.0%) who were premature. Importantly, among the LBW deliveries, only 12 (24%) were premature, such that most LBW was due to IUGR, rather than prematurity. Given the relatively small number of newborns who were premature-SGA (n = 7) or premature-LBW (n = 12), we did not analyze these sub-groups separately. Between one and six-months-old, there were significantly more non-LBW infants (93.2%) who were exclusively breastfed than LBW infants (84.0%).

**Table 1:** Basic descriptive data by birthweight and size for gestational age^a^.

Covariate	Birthweight			Size for gestational age		
	Low birthweight^b^ (n = 50)	Non-low birthweight (n = 307)	*P* value^c^	Small-for-gestational age^d^ (n = 82)	Non-small-for-gestational age (n = 275)	*P* value
Birth data						
Female, %	44.0 (30.2–57.8)	47.2 (41.6–52.8)	0.67	40.2 (29.6–50.8)	48.7 (42.8–54.6)	0.17
Length, cm	44.9 (44.0–45.8)	47.2 (46.8–47.5)	<0.001	46.0 (45.4–46.7)	47.1 (46.7–47.5)	<0.001
Weight-for-length	0.049 (0.047–0.051)	0.063 (0.062–0.064)	<0.001	0.053 (0.052–0.055)	0.063 (0.062–0.064)	<0.001
Weight, kg	2.19 (2.09–2.28)	2.96 (2.93–3.00)	<0.001	2.45 (2.38–2.51)	2.98 (2.93–3.02)	<0.001
Gestational age, wk	37.5 (36.8–38.2)	38.7 (38.6–38.8)	<0.001	39.0 (38.7–39.3)	38.4 (38.2–38.6)	<0.001
Small-for-gestational age, %	80.0 (68.9–91.1)	20.0 (15.5–24.5)	<0.001			
Low birthweight, %				48.8 (38.0–59.6)	3.6 (1.4–5.8)	<0.001
Feeding						
Exclusively breastfeeding, %						
Birth to 1month	90.0 (81.7–98.3)	94.5 (91.9–97.1)	0.21	90.2 (83.8–96.6)	94.9 (92.3–97.5)	0.12
1 to 6 months	84.0 (73.8–94.2)	93.2 (90.4–96.0)	0.045	86.6 (79.2–94.0)	93.5 (90.6–96.4)	0.046
6 to 12 months	82.0 (71.4–92.6)	90.6 (87.3–93.9)	0.07	85.4 (77.8–93.0)	90.6 (87.2–94.0)	0.18
First-trimester maternal data						
Age, y	25.3 (23.2–27.4)	26.2 (25.5–26.9)	0.19	26.0 (24.5–27.6)	26.1 (25.3–26.8)	0.76
Parity, no	3.1 (2.5–3.8)	3.7 (3.4–3.9)	0.027	3.4 (3.0–3.9)	3.7 (3.4–3.9)	0.31
Height, cm	146.6 (145.2–148.0)	147.6 (147.0–148.2)	0.26	146.1 (144.9–147.3)	147.9 (147.2–148.5)	0.011
Body mass index, kg/m^2^	21.7 (20.8–22.5)	21.9 (21.6–22.3)	0.56	21.9 (21.3–22.5)	21.9 (21.5–22.2)	0.77
Weight, kg	46.6 (44.7–48.5)	47.7 (46.9–48.5)	0.28	46.7 (45.3–48.0)	47.8 (46.9–48.7)	0.34
Education (≥ high school)	66.0 (52.9, 79.1)	56.4 (50.9, 62.0)	0.20	57.3 (46.6–68.0)	57.8 (52.0–63.6)	0.94

^a^Values are means or proportions (95% confidence intervals), n = 357.

^b^Defined as birthweight <2.5kg.

^c^Tested by Student’s t test or Wilcoxon’s rank-sum test for continuous variables and Fisher’s exact or Chi-square test for categorical variables.

^d^Defined as birthweight <10th percentile for gestational age.

**Fig 2:**
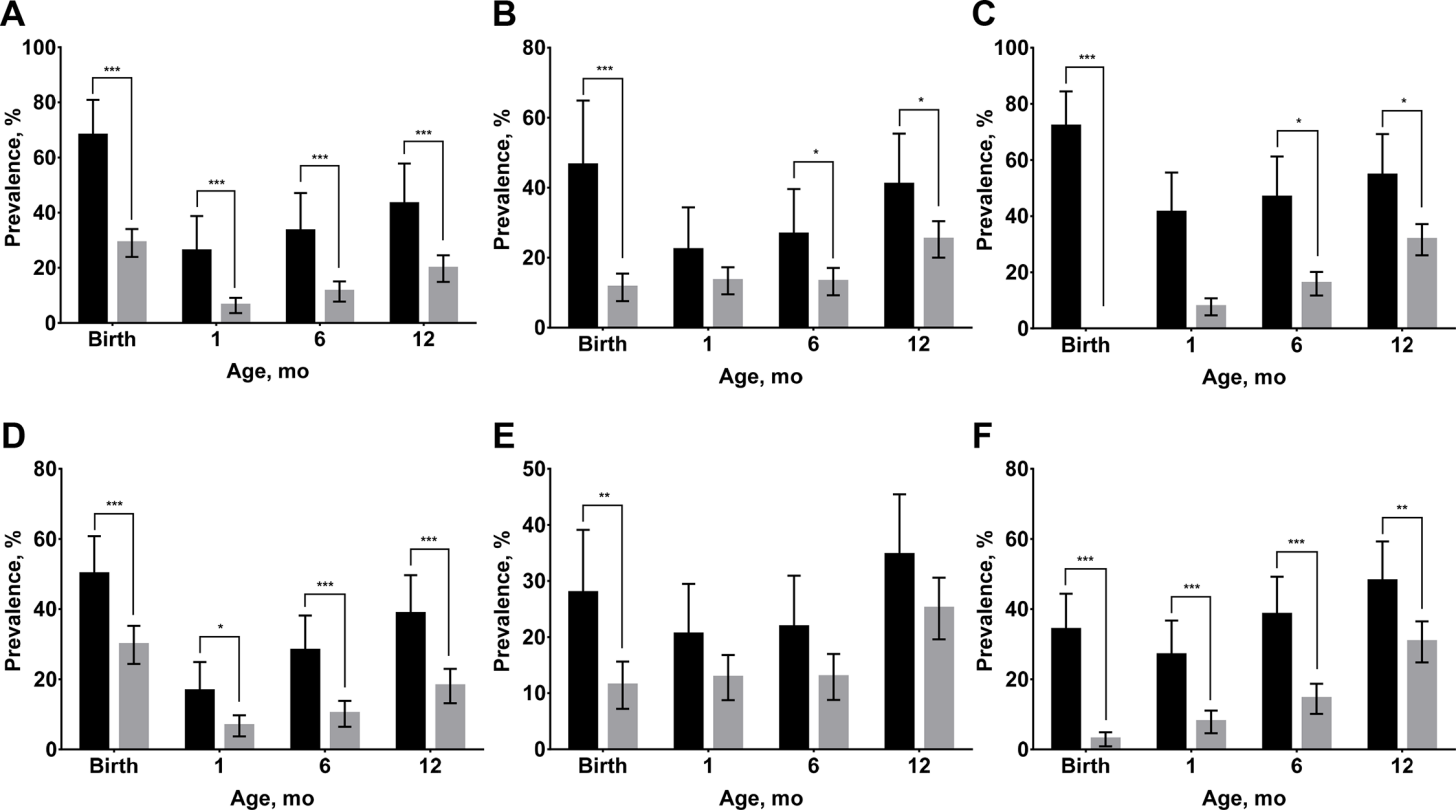
**Stunting, wasting, and underweight from birth to 12 months of age by birthweight (A, stunting; B, wasting; C, underweight) and size for gestational age (D, stunting; E, wasting; F, underweight).** Values are prevalence and 95% confidence intervals, n = 357. In A, B, and C, black bars indicate low birthweight; gray bars indicate non-low birthweight. In D, E, and F, black bars indicate small-for-gestational age; gray bars indicate non-small-for-gestational age. Stunting, wasting, and underweight were defined as height-for-age Z score <-2.0, weight-for-height Z score <-2.0 and weight-for-age Z score <-2.0, respectively. Low birthweight was defined as birthweight <2.5kg. Small-for-gestational age was defined as birthweight <10^th^ percentile for gestational age. *P <0.05, ** *P* <0.01, *** *P* <0.001 different from low birthweight or small-for-gestational age group.

**Fig 3:**
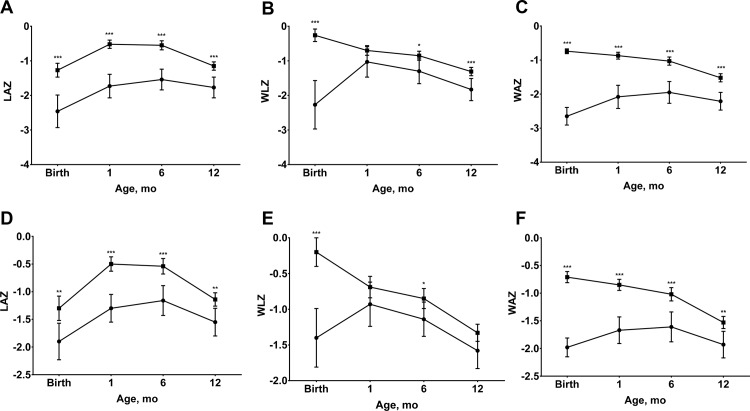
**Length-for-age (LAZ), weight-for-length (WLZ) and weight-for-age (WAZ) z scores from birth to 12 months of age by birthweight (A, LAZ; B, WLZ; C, WAZ) and size for gestational age (D, LAZ; E, WLZ; F, WAZ).** Values are means and 95% confidence intervals, n = 357. In A, B, and C, square symbols indicate low birthweight; circle symbols indicate non-low birthweight. In D, E, and F, square symbols indicate small-for-gestational age; circle symbols indicate non-small-for-gestational age. Low birthweight was defined as birthweight <2.5kg. Small-for-gestational age was defined as birthweight <10^th^ percentile for gestational age. *P <0.05, ** *P* <0.01, *** *P* <0.001 different from low birthweight or small-for-gestational age group.

At each age, significantly more LBW infants remained stunted and underweight compared to non-LBW infants and the same patterns were observed among SGA and non-SGA infants (Fig 2). Significantly more LBW infants were wasted at birth, six and 12-months-old compared to non-LBW infants, however, the only significant difference in the prevalence of wasting between SGA and non-SGA infants was observed at birth. Similarly, significant differences were found at most timepoints for mean LAZ and WAZ between LBW and non-LBW infants and between SGA and non-SGA infants (Fig 3). The only exception was the convergence of mean LAZ for SGA and non-SGA infants by 12-months-old (Fig 3D). Non-LBW infants had significantly higher WLZ than LBW infants at birth, six and 12-months-old, while differences in WLZ between SGA and non-SGA infants were found at birth and six-months-old.

**Fig 4:**
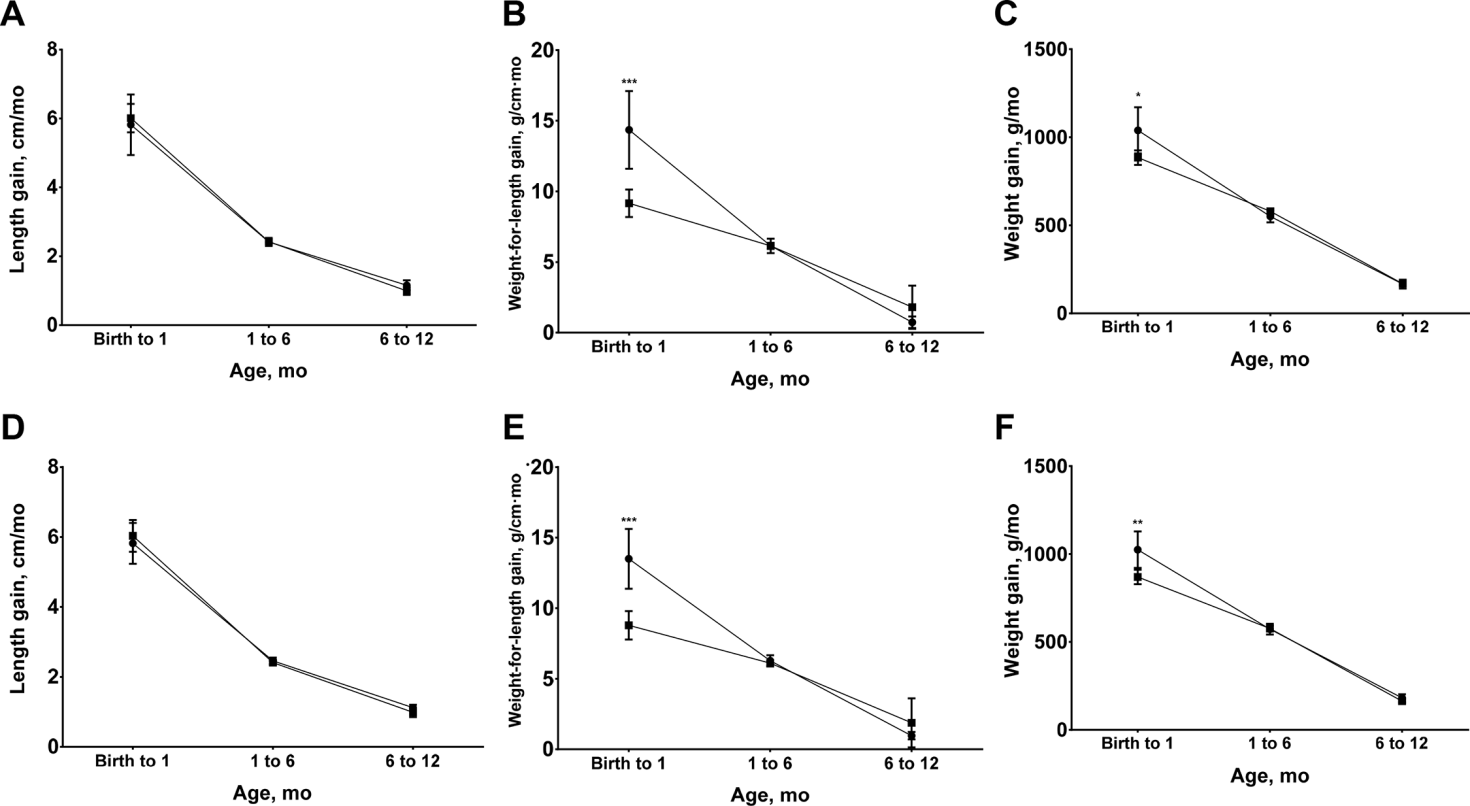
**Length, weight-for-length and weight gains from birth to 12 months of age by birthweight (A, length gain; B, weight-for-length gain; C, weight gain) and size for gestational age (D, length gain; E, weight-for-length gain; F, weight gain).** Values are means and 95% confidence intervals, n = 357. In A, B, and C, square symbols indicate low birthweight; circle symbols indicate non-low birthweight. In D, E, and F, square symbols indicate small-for-gestational age; circle symbols indicate non-small-for-gestational age. Low birthweight was defined as birthweight <2.5kg. Small-for-gestational age was defined as birthweight <10^th^ percentile for gestational age. *P <0.05, ** *P* <0.01, *** *P* <0.001 different from non-LBW or non-SGA group.

The significantly higher weight-for-length gains of LBW and SGA infants between birth and one month of life (Fig 4) diminished differences in the prevalence of wasting (Fig 2B and 2E) and reduced differences in WLZ after one-month-old (Fig 3B and 3E). Significant, but small differences in weight gain in the neonatal period, however, did not fully mitigate the significant differences in the prevalence of underweight infants (Fig 2C and 2F) among LBW and SGA newborns and differences in WAZ (Fig 3C and 3F). This is likely due to the fact that WAZ captures both linear growth faltering and wasting. Finally, neither LBW nor SGA infants experienced higher linear growth velocities (Fig 4A and 4D), such that infants in these disadvantaged birth categories had persistently higher prevalence of stunting and lower mean HLZ throughout infancy (Figs 2 and 3A and 3B).

In the univariable GEE models, the odds of stunting throughout infancy were significantly increased when infants were male, LBW, SGA and bottle or mixed fed versus exclusively breastfed (Table 2). The odds of stunting were decreased when infants had a higher GA at birth and when mothers were taller. The probability of wasting was increased for infants who were LBW, SGA or had mothers who were older or had lower educational attainment. Underweight was significantly associated with the infant’s sex, GA and LBW and SGA status, as well as with maternal age, height and weight.

**Table 2:** Univariable generalized estimating equations models predicting stunting, wasting and underweight at three time points during infancy^a^.

Covariate	Reference	Stunting^b^		Wasting^c^		Underweight^d^	
		OR (95% CI)	*P* value	OR (95% CI)	*P* value	OR (95% CI)	*P* value
Sex (male)	Female	1.78 (1.12, 2.85)	0.015	1.27 (0.88, 1.83)	0.20	2.07 (1.37, 3.11)	<0.001
Gestational age, wk		0.69 (0.61, 0.79)	<0.001	0.98 (0.85, 1.14)	0.81	0.70 (0.62, 0.80)	<0.001
Low birthweight (LBW)^e^	Non-LBW	3.82 (2.29, 6.37)	<0.001	2.12 (1.35, 3.33)	0.001	4.30 (2.58, 7.16)	<0.001
Small for gestational age (SGA)^f^	Non-SGA	2.98 (1.88, 4.72)	<0.001	1.73 (1.16, 2.58)	0.008	2.90 (1.88, 4.47)	<0.001
Feeding (breastfeeding or bottle formula)	Exclusively breastfeeding	2.30 (1.38, 3.81)	0.001	0.97 (0.53, 1.76)	0.92	1.46 (0.86, 2.46)	0.16
Maternal age (≥30 y old)	<30 years old	1.05 (0.65, 1.71)	0.83	1.71 (1.18, 2.49)	0.005	1.63 (1.09, 2.44)	0.018
Maternal height, cm		0.95 (0.92, 0.99)	0.007	0.99 (0.96, 1.03)	0.59	0.95 (0.91, 0.99)	0.008
Maternal body mass index, kg/m^2^		0.98 (0.91, 1.05)	0.56	0.97 (0.91, 1.04)	0.44	0.97 (0.91, 1.03)	0.34
Maternal weight, kg		0.97 (0.94, 1.00)	0.035	0.98 (0.96, 1.01)	0.22	0.96 (0.94, 0.99)	0.008
Maternal education (<high school)	≥high school	1.16 (0.74, 1.80)	0.52	1.50 (1.04, 2.16)	0.031	1.43 (0.96, 2.12)	0.08

^a^Values are odds ratios (ORs) [95% confidence intervals (CIs)], n = 357.

^b^Defined as length-for-age Z score <-2.0.

^c^Defined as weight-for-length Z score <-2.0.

^d^Defined as weight-for-age Z score <-2.0.

^e^Defined as birthweight <2.5kg.

^f^Defined as birthweight <10^th^ percentile for gestational age.

In the multivariable GEE analyses (Table 3), the final model with LBW as the primary predictor for risk of stunting during infancy retained the following covariates; sex, GA, LBW, feeding type, and maternal height. The stunting model with SGA included sex, SGA, feeding type, and maternal height. The two distinct final models with LBW or SGA for the outcome of wasting included the same confounders, maternal age and maternal educational attainment. The model for underweight with LBW included sex, GA, LBW, and maternal age, while the SGA model included sex, and maternal age. Also of note, parity and maternal age were highly correlated such that we could not retain both in the final models. Parity, however, was significantly related to both wasting and underweight when maternal age was removed from the model (data not shown). There were no significant interactions between either birthweight category and other predictors.

**Table 3:** Multivariable generalized estimating equations models predicting stunting, wasting and underweight during infancy^a^.

Covariate	Reference	Stunting^b^		Wasting^c^		Underweight^d^	
		OR (95% CI)	*P* value	OR (95% CI)	*P* value	OR (95% CI)	*P* value
**Low birthweight (LBW) Model**							
Sex (male)	Female	2.06 (1.24, 3.42)	0.005			2.21 (1.41, 3.45)	<0.001
Gestational age, wk^e^		0.72 (0.63, 0.83)	<0.001			0.74 (0.65, 0.85)	<0.001
LBW^f^	Non-LBW	2.64 (1.53, 4.53)	<0.001	2.33 (1.47, 3.70)	<0.001	3.76 (2.21, 6.40)	<0.001
Feeding (breastfeeding or bottle formula)	Exclusively breastfeeding	2.46 (1.44, 4.20)	0.001				
Maternal age (≥30 y old)	<30 years old			1.73 (1.18, 2.52)	0.005	1.88 (1.23, 2.86)	0.003
Maternal height, cm		0.95 (0.91, 0.99)	0.007				
Maternal education (<high school)	≥high school			1.56 (1.08, 2.27)	0.019	1.52 (1.01, 2.31)	0.047
**Small for gestational age (SGA) Model**							
Sex (male)	Female	1.81 (1.13, 2.89)	0.014			2.01 (1.33, 3.04)	0.001
SGA^g^	Non-SGA	2.60 (1.62, 4.19)	<0.001	1.75 (1.16, 2.64)	0.007	2.82 (1.81, 4.37)	<0.001
Feeding (breastfeeding or bottle formula)	Exclusively breastfeeding	2.27 (1.36, 3.79)	0.002				
Maternal age (≥30 y old)	<30 years old			1.70 (1.17, 2.48)	0.006	1.73 (1.15, 2.61)	0.009
Maternal height, cm		0.95 (0.92, 0.99)	0.017				
Maternal education (<high school)				1.47 (1.02, 2.13)	0.040		

^a^LBW and SGA models were built with low birthweight and small-for-gestational age as primary predictor respectively. Values are odds ratios (ORs) [95% confidence intervals (CIs)], n = 357. Maternal height was considered in the multivariable model for stunting, and maternal body mass index for wasting and underweight.

^b^Defined as length-for-age Z score <-2.0.

^c^Defined as weight-for-length Z score <-2.0.

^d^Defined as weight-for-age Z score <-2.0.

^e^Evaluated only for LBW model.

^f^Birthweight < 2.5 kg.

^g^Defined as birthweight <10^th^ percentile for gestational age.

## Discussion

Our results suggest that LBW and SGA infants in this setting do not catch up to non-LBW and non-SGA infants, even by age 12 months, with respect to most measures of linear growth and nutritional status. A slightly greater weight gain among both LBW and SGA infants was limited to the first month of life, explaining some convergence in WAZ and WLZ by one–month-old, but with little catch up thereafter. The lack of any significant differences in linear growth velocity during any window led to significant difference in risk of stunting and LAZ throughout infancy. This differential growth pattern is likely due to rapid soft tissue gain in the first few months of life, that did not, however, allow catch up growth [17]. These findings contrast those conducted in industrialized nations, where a large proportion of infants born SGA achieve weight and length catch up growth during infancy [18–20]. This is likely due to the availability of human milk fortifiers, the availability of formulas with higher kilocalories per ounce, and maternal nutritional status, with better nourished mothers providing greater volume of breast milk with higher fat content [21, 22]. In addition, early weaning to complementary foods with low protein and fat content, such as rice, may also hinder catch up growth.

The persistently increased odds of growth stunting and undernutrition in LBW and SGA infants that we observed are consistent with studies conducted in other resource-constrained settings in Africa and Asia [3, 23–26]. In a cohort study in Cebu, The Philippines, LBW was a predictor of stunting until at least two years of age [3]. In a separate study in Metro Cebu, LBW status increased the odds of stunting at six and 12-months-old [25]. A Tanzanian cohort found that newborns with birthweight <10^th^ percentile had over twice the risk of stunting and 1.45 times the risk of wasting compared to the other newborns throughout the first 18 months of life [24].

Importantly, studies in the United States have shown that IUGR and SGA infants have lower nutritional Z-scores during infancy and early childhood than infants with the same birthweight who were born prematurely [9, 27–30]. This suggests that prematurity results in a less permanent growth impairment than IUGR, with the latter process beginning *in utero* [27]. These findings are especially relevant to infants in LMIC settings, where a greater proportion of LBW deliveries are consequent to IUGR than prematurity, though this is difficult to determine with certainty due to limitations of GA determination in this setting [9, 31]. Given this, and the fact that 80% of the LBW newborns in this cohort were SGA, in the LMIC setting LBW status likely captures a significant proportion of SGA newborns who are more easily identified and remain at risk.

In addition to LBW and SGA, our study identified other risk factors for stunting, wasting and underweight. Breastfeeding was shown to be a protective factor for decreasing odds of stunting among all infants regardless of birthweight status. This is consistent with the well-described benefits of exclusive breastfeeding in Filipino infants and other infants in LMIC settings [8, 25, 32–35]. This is likely due to poor nutritional value of supplementary foods in these regions and lack of clean water sources for formula, which increases the risk of diarrheal illness and other infections [36]. Importantly, already at risk LBW infants were somewhat less likely to be exclusively breastfed, which supports the findings of previous studies in Cebu [37].

Male sex was also a significant risk factor for stunting in LBW infants and for underweight in LBW and SGA infants. In previous studies in Filipino infants, males were more likely to become stunted in the first year of life, and females in the second year of life [3]. Males may be more susceptible to impairments in growth in early life due to their more rapid growth trajectory than girls over this period, which is likely the result of a sex-specific epigenetic process that begins *in utero* [18, 38, 39]. As expected, maternal height was a risk factor for stunting in both LBW and SGA infants. Similarly, previous studies in LMIC settings describe maternal short stature as a predictor for both LBW and stunting during infancy [40–43]. Maternal age (≥30 years) was a risk factor for wasting and underweight in both groups, yet not for stunting. By contrast, a recent study showed that extremes of low (<30 years) and high (>45 years) maternal age increases the risk of stunting during infancy [44]. Importantly, age may also be capturing the effects of parity, as we found that parity was significantly related to risk of wasting and underweight when maternal age, with which it was highly correlated, was removed from models. This is likely due to greater food insecurity for mothers and the infant participants in homes with more children,

Importantly, other studies of LBW infants in the Philippines have reported that postnatal growth patterns are significantly related to social economic status [25]. Our results show that lower maternal educational attainment was a risk factor for undernutrition during infancy, particularly wasting. This emphasizes the key role of maternal education in addition to the key public health messaging needed to encourage exclusive breast feeding, as suggested by other studies [8, 17].

Though previous studies have examined growth and nutritional outcomes in the LMIC setting, this study adds a determination of SGA that is more likely to be accurate based on the use of 12–16 week ultrasound for GA determination. The use of the INTERGROWTH-21^th^ rather than Western-based reference curve for SGA also makes it more likely that these SGA infants experienced IUGR. In addition, the longitudinal design allowed for better assessment of causality in determining risk factors for undernutrition and growth faltering during infancy.

Limitations of this study include the fact that mothers and infants were from a rural region of The Philippines, which may limit generalizability. In addition, though we considered SGA births as newborns who likely experienced IUGR during pregnancy, it is possible that some of these newborns, in fact, reached their *in utero* growth potential and did not experience IUGR. As above, use of a healthy reference curve comprised of newborns from multiple different nations, including LMICs, mitigates this concern somewhat. With respect to etiology of SGA, limitations with respect to our ability to diagnose infections, particularly viral infections that might impact growth *in utero*, preclude identifying these common etiologies. In addition, the definition of pre-eclampsia employed at the time of study inception required two elevated blood pressures separated by four hours and most refused to wait. Though many women were diagnosed at delivery with pre-eclampsia based on elevated blood pressure, lack of application of standard definition did not allow us to determine the percent of newborns who were SGA due to this very common etiology. Finally, these infants were only followed until 12-months-old, however, additional studies of these children at five years of age will further elucidate long-term growth and nutritional catch up.

Though LBW and SGA infants in this setting exhibit increased weight and weight-for-length velocity in the first month of life, they remain at significantly higher risk of undernutrition and do not catch up to non-LBW and non-SGA infants by 12-months-old. Importantly, infants who were LBW were actually less likely to be exclusively breastfed at six-months-old. Lower maternal educational attainment continues to influence the risk of undernutrition in this setting among all infants, emphasizing the need for education regarding exclusive breastfeeding and health literacy in order to the decrease the risk of stunting and undernutrition in these high risk groups.
